# Mechanisms of Timing, Timbre, Repertoire, and Entrainment in Neuroplasticity: Mutual Interplay in Neonatal Development

**DOI:** 10.3389/fnint.2020.00008

**Published:** 2020-03-02

**Authors:** Joanne Loewy, Artur C. Jaschke

**Affiliations:** ^1^The Louis Armstrong Center for Music and Medicine, Mount Sinai Beth Israel, Icahn School of Medicine, New York, NY, United States; ^2^Department of Music Therapy, Beatrix Children’s Hospital—University Medical Centre, ArtEZ University of the Arts, Groningen, Netherlands; ^3^Department of Neonatology and Clinical Neuropsychology, Amsterdam, Netherlands

**Keywords:** music plasticity, neural music mapping, neonatal music mapping, music brain, neonatal music therapy

## Abstract

Neonatal brain development relies on a combination of critical factors inclusive of genetic predisposition, attachment, and the conditions of the pre and postneonatal environment. The status of the infant’s developing brain in its most vulnerable state and the impact that physiological elements of music, silences and sounds may make in the earliest stages of brain development can enhance vitality. However, little attention has been focused on the integral aspects of the music itself. This article will support research that has hypothesized conditions of music therapeutic applications in an effort to further validate models of neurobehavioral care that have optimized conditions for growth, inclusive of recommendations leading toward the enhancement of self-regulatory behaviors.

## Introduction

Recent research has evaluated the effects of music training with benefits that have attributed music learning as a significant contributor to the improvement of cognitive function (Barrett et al., 2013). In particular, music’s implicit audio-structural capacity (Lordier et al., 2019) seems to reflect notable influence in both developmental outcomes and restorative capabilities that stretch across the lifespan. While numerous studies have focused on outcomes of young children (Trainor et al., 2012; Moreno et al., 2011) and aging adults (Johnson et al., 2011), few have considered the impact of the pre-natal ‘sound’ environment in terms of ritual and context, nor the culturally relevant conditions and applications that organized purposeful sounds and music may stimulate. Elements of sound in and out of music and their structured or disorganized contexts can be considered as disruptive or as viable enhancements to the advancement of the Neonatal Intensive Care Unit (NICU) experience (Stewart and Schneider, 2000; Rossetti and Canga, 2013), particularly as the conditions of the infant’s brain in its most vulnerable state are at stake. The impact that the physiological elements of music, silences and sounds, can make in the earliest stages of brain development is worth consideration. This article will support research that has hypothesized conditions of music therapeutic applications validating models of neurobehavioral care that have provided proven optimal conditions for growth inclusive of recommendations leading toward the enhancement of self-regulatory behaviors. Developmental markers based on research and clinical experiences related to current quantitative data of musical elements have been sparse. Clinical experiences substantiated through the First Sounds: Rhythm, Breath and Lullaby research (Loewy et al., 2013) that lead to a model of practice and training will be explicated within this article (First Sounds, 2016). Specifically, we will address the optimal contexts for the essential foundational relationships between infants and caregivers based on music’s neural plasticity factors. These can be enhanced through attachment conditions which are fortified through music to address its relationship in developing executive functions (EFs) across the lifespan (Sala and Gobet, 2017). Elements contributing to this context include live vital sign entrainment, social interaction *via* attunement, rhythmic assessment with the institution of indicated repertoire, and sleep/wake patterns inclusive of aural transition capacity.

## Factors of Influence

### Genetic Predisposition

Multiple influences such as auditory, visual or somatosensory factors, show a trend in influencing brain dynamics in young clinical populations, and especially in prematurely born infants in the NICU. These factors have the tendency to translate into later childhood and even adulthood expression (Galvan, 2018). Some of these trends can be seen as factors of influence in cognitive and overall neural development. Neural differences in social cognition, which can be triggered by hormonal changes, or by experiences of an “unnatural” sensory environment to the infant, have been described (Galvan, 2018). Their influence requires more research, as larger cohort studies are still scarce.

Neonatal growth relies on a combination of critical factors that contribute to brain development. In our experience of studying neonates’ responses to music therapy stimuli, and in working within dyads and triads involving father, mother, staff and neonate, the factors of influence where music’s potential might enhance developmental prowess are worth defining. This is because the neonate’s sensitivity to sound and music is distinctly and definably related to neurologic outcomes. These factors are inclusive of genetic predisposition (Montirosso, 2015) and the conditions of the pre and postneonatal environment. There are distinct periods of infant development whereby a genetically determined microcircuitry of key limbic-hypothalamic-midbrain structures are particularly sensitive to early environmental contexts. These influences directly formulate an individual’s responsiveness to psychosocial stressors as well as the resiliency or susceptibility to difficult circumstances in later life circumstances (Leckman et al., 2004).

### Caregiver Capacity for Secure Attachment and Aural Sound Conditions

Developmental factors are typically attributed to a caregiver’s knowledge and capacity for creating opportunities for secure attachment (Lomanowska et al., 2017). Emerging research undertaken first by Bowlby (Bretherton, 1992) and subsequently his student, Ainsworth emphasized the significance of secure attachment, and its relationship as a most critical interplay between the biological and environmental conditions of two systems, that of the mother (parent) and of the infant (Bretherton, 1992; Zimmer, 2019).

This interplay, coupled with an aural sensitivity (Lahav and Skoe, 2014) within a context conducive to optimal sound conditions can foster neural organization. Aural sensitivity is the intricate perception and processing of sounds. These cover a variety of different frequencies and can be traced back to, however not exclusively to, the tonotopic map of the primary auditory cortex. Moreover, sensitivity to sound reaches further than the primary auditory cortex and can have implications beyond the temporal lobe resulting in changes in behavior (avoidance, interest) or emotional responses (e.g., pain).

One study used a repeated-measures research design to examine variables of neonates recorded every 2 min through two 60-min observation periods for each research day (1 h in the morning and 1 h in the afternoon) over 2 days. Thirty-seven preterm infants provided a total of 4,164 observations that were made rendering a statistically significant (*P* < 0.05) relationship between environmental stressors and changes in physiological signals. There were also statistically significant (*P* < 0.05) relationships between environmental stress and specific stress behaviors. Physiologic stress responses to acoustic events such as changes in heart rate, intracranial pressure, and oxygen saturation may have a significant impact on the preterm infant’s future neurologic development due to altered perfusion and oxygenation of the brain tissue (Peng et al., 2009). These changes have been shown to influence neurologic development. This study has shown a significant correlation between environmental stressors and physiological signals of bio-dynamical changes.

### Trauma and Pain

Another factor that has moved in and out of the literature base encasing elements respective to the aforementioned conditions, is related to the concept of trauma. There is a suspected neurobiological mechanism by which maternal unresolved trauma can modify maternal caregiving so as to disrupt the normative development of an infant. This was highlighted in a recent review culminating a plethora of research indicating the impact of childhood adversity and its effects on parenting, which may be perpetuated inter-generationally (Kim et al., 2018).

Music therapist Kristen Stewart developed a model for treating trauma with premature infants and parents-entitled: Preventative Approach to Traumatic Experience by Resourcing the Nervous System (PATTERNS). Instituting music therapy in a preventative treatment model that is based on latent human resiliency and trauma renegotiation principles, Stewart’s model (Stewart, 2009a) involves integrative mechanisms that influence perception, nurturance, and opportunities for building resilience at all levels within—infant-parent—and staff contexts utilizing music therapy.

An additional factor that affects neonatal neural activity involves the distinct formation of neuronal connections, including dendritic spine development, synaptogenesis, axon myelination, and crucial cortical folding of the neocortex (Dubois et al., 2008). Importantly, developmental changes are not wholly determined by intrinsic prenatal biological factors, but by postnatal experiences as well. While axonal guidance and myelination, and axonal elements are thought to be influenced by genetics, the distinct pathways of connections that are retained rather than pruned are within the axon myelination process as well and are sensitive to the sound environment (Benasich and Ribery, 2018). This brings to the forefront of the realization that neonatal neural activity is affected by pain (Anderson et al., 2011). The younger the gestational age of a premature infant’s receptive fields are the more vulnerable the effects of pain and in this way, the result of pain can involve hypersensitivity (Fitzgerald and Walker, 2009). Since it is known that sensory afferents are not “hard-wired,” but plastic (Ren and Dubner, 2007) and given the wider receptive fields and hypersensitivity of preterm neonates, the pain they experience in this vulnerable time may have profound long-lasting effects. Primary hypersensitivity is more easily provoked in preterm neonates (Johnson et al., 2011). Attention to the neonates’ early experience of pain has ramifications for plasticity and neural development. A recent study utilized live lullaby singing (song of kin-as selected from parental preference), for the first time on behavioral and psychological pain responses during venipuncture of 38 preterm and full-term infants with some interesting findings towards higher oxygen saturation and calmer heart rates in the intervention groups as compared to the control group. This could be an important development for future neonatal and young infant pain protocols (Ullsten et al., 2017).

### Pre-mature Brain Function

Our knowledge of brain functions during the perception and processing of music has increased in the past decade (for review, see Jaschke, 2019). Modern technological advances such as functional Magnetic Resonance Imaging, Diffusion Tensor Imaging, and functional Near Infrared Spectroscopy, have made it possible to track and analyze music multisensory stimulus as it activates several brain regions in its path (Jaschke, 2019). Music is comprised of complex stimuli that activate regions beyond the auditory cortex. Among others it activates the thalamus, hippocampus, and temporal as well as frontal regions; all of which are known to have additional primary functions such as language processing, EFs, and associative memory.

Recent developments in music and brain research justify music as a multi-sensory stimulus and the influence of its multisensory integrative properties on dynamic brain networks (Barrett et al., 2013). There is still more research needed to investigate these properties in neurotypical pediatric as well as adult brain function. Multisensory properties are even less studied in the neonatal/preterm brain and will certainly assist in our potential to develop formative hypotheses that may well have bearing on all aged populations. Neonatal brain access allows for the study of neural plasticity in light of multisensory integration in real-time.

The multiple brain areas involved in the perception and processing of music make it a potentially interesting clinical tool in NIC (Sa de Almeida et al., 2020). Throughout the development of the brain, coupling and synchronization of different brain areas are thought to mediate between the assembly and development of neural networks, which in turn contribute to the coordination and plasticity of the brain as well as play supporting roles in sensorimotor functions such as cognition including perception, language, learning, executive functioning and memory (Benasich and Ribery, 2018; Sa de Almeida et al., 2020).

Concerning sensorial influences in the NICU, a specific interrupted process weighs upon auditory brain development, which starts early in gestation. By 23–25 weeks, all structures necessary for hearing, among which the cochlea, have developed (McMahon et al., 2012) As such, most infants admitted to the NICU are able to hear, unless they have a congenital anomaly.

From approximately 26 weeks’ gestation, fetuses or preterm infants will have the capacity to react to auditory stimuli. Sounds a fetus hears within the womb include a mother’s heartbeat, respiration and the maternal voice, and show recognition to the mother’s voice, and in some circumstances, father’s voice as well (Lee and Kisilevsky, 2014). From 30 weeks’ gestation onward, the infant is able to distinguish between varying speech tones and timbres and is also able to process complex auditory sounds. This point likely marks the start of speech and language development (McMahon et al., 2012).

In preterm birth, this process is interrupted. On the one hand, this means that infants are exposed to noise in the NICU that they may not yet be able to process, which could be harmful and has been shown to alter respiratory and cardiac functions (Sa de Almeida et al., 2020). On the other hand, the deprivation of sounds heard *in utero* could have consequences for auditory brain maturation affecting speech and language development. A recent study of 136 (<30 weeks) infants hospitalized in a quiet private room NICU environment vs. an open active ward showed lower language and motor scores and a trend towards lower cerebral maturation at follow up- 2 years of age (Pineda et al., 2014). In a second study, a literature review of 88 studies inclusive of measurements related to the auditory environments in the NICU sought to determine medical, environmental, and socio-demographic factors that predict and further define influential protagonists and antagonists related to infant auditory exposure in the NICU.

This review stratified succinct elements of the sounds in both conditions, the open and private room NICU-such as Noise, Mechanical equipment, meaningful words, electronic noise, adult words, distant words, et cetera (Pineda et al., 2017). It was found that there was an average of 3 h more silence in a 16-h period in the private room-that equated to roughly 30% more silence than in the open ward. This study, among other reports (Jobe, 2014; Webb et al., 2015) makes a case for the importance of low-frequency sound exposure for neonates. At the same time recommendations on the importance of all NICU sound environments whether in open or private spaces should seek to define the specific rationale for implementing any specific modality, inclusive of the timing and frequency considerations of sensory-based interventions. The goal should include strategies that optimize and inform any environmental modifications.

Examining infant sound exposure and seeking opportune experiences for premature infants inclusive of music will provide assurances that they will have exposure to meaningful words (McGrath, 2013), inclusive of language or singing that is infant-directed. Sound making and holding can also provide optimal conditions for development (Teckenberg-Jansson et al., 2011).

This delicate balance of activity, inhibition and multimodality integration prompts our capacity to make sense of the world around us in an attempt to perceive through multi-signaling. These dynamic processes develop over time through learning, exposure, and experience existent through until adulthood and beyond (Freeman, 1997). Such mechanisms are part of a complex and demanding process, which can place a fair amount of strain on any neurotypically developing infant, child or adult. Neonates and especially preterm born children face aside from the aforementioned difficulties, additional challenges as their brains and bodies encounter the complexity of these developing networks prematurely (Arnon et al., 2006).

After birth, the brain not only develops as a result of internally driven mechanisms alone. Neural circuitry is shaped by external factors and biogenetical mechanisms as well. The impact of external factors on a not-yet-ready-brain can, therefore, have a significant impact on the development of a child during both childhood and adulthood (Bieleninik et al., 2016; Provenzi et al., 2018). Considerations taken between over and under stimulation when it comes to understanding and supporting the development of a prematurely born infant brain have been scrutinized historically and currently. In order to be able to stimulate the brain to provide the infant with the best possible start; sounds and voices can be used as therapeutic stimuli (Loewy, 2015). The signature sound of the womb is one that an early pioneer, Van de Carr and Lehrer (1988) examined and notoriously named a “prenatal university.” These sounds are thought to be the most critical aspect of co-regulation for the neonate and mother, and subsequently family and community adaptation (Van de Carr and Lehrer, 1988). The mother’s heart rhythm, and her voice pitch, tone and rhythm are hallmarks of an infant’s first auditive “memory” prior to birth (Webb et al., 2015). Taking this a step further, in pro-active music activity with infants, exaggerated speech, such as singing, where the tones, pitches and intervals are purposefully arranged and orchestrated to meet the expression of the neonates, enhanced responsiveness develops. The inclusion of fathers is advisable. Breathing rhythms and tonal/vocal expressions become an active means of co-regulation and form the basis of expressive language and emotional attachment.

Stimulating the premature brain in this stage with entrained sounds, language, or music has to be implemented and coordinated with conscious effort and rationale, as the cortical folding is still in flux and a great number of neurons are still migrating to their final cortical destinations (Provenzi et al., 2018). Applied cautiously and consciously, parental and therapeutically trained voices and music can enhance neural development and stimulate neural plasticity, which in turn has the potential to influence cognitive function, brain oscillation and stimulate dynamic brain plasticity and network formation, in short as well as long-term development (Bieleninik et al., 2016; Provenzi et al., 2018; Sa de Almeida et al., 2020).

### Music and Music Therapy as Neural Stimulation

Early in gestation—at around 16 weeks, the fetus’s auditory system is formed. Between the 26th and 30th weeks, the fetus is able to detect and react to sound stimuli. This period in the womb is considered to be a critical period for neurodevelopment (Benasich and Ribery, 2018). Vandormael et al. (2019) discuss how the “fine-tuning process takes place in the uterus where both internal (e.g., respiration, heart rhythm, and digestion) and external sounds (e.g., voices and music) can be perceived” (Vandormael et al., 2019). They also cite significant recent research in numerous NICU conditions showing evidence that too much chaotic noise or not enough sounds, largely people’s voices, may have detrimental, or deprived conditions which can show up in delayed language capacity in toddlerhood.

In a recent study, of 272 infants born prematurely, and serving as their own controls within a randomized 2-week intervention period in 11 hospitals, when exposed to entrained music therapy conditions, either in parental or music therapist singing conditions, vital signs improved. Implementing song of kin; parent-selected familiar melodies, or improvised to create in-the-moment, meaningful lyrics, within a simple melodic construct, or using womb sounds simulated through the use of a quietly entrained Remo ocean disc, sleep patterns were enhanced, and heart rate patterns were more even (regulated) and soothed (promoting sleep; Loewy et al., 2013).

The implementation of entrainment conditions, confirm and substantiate that live music in a music therapy context is best indicated. Live music ensures feedback that makes for reliable patterning of a sound-based relationship and a safe environment as music is regulated, in real-time, at the moment. The implementation of entrainment conditions, confirm and substantiate that live music in a music therapy context is best indicated. Live music ensures feedback that makes for reliable patterning of a sound-based relationship and a safe environment as music is regulated, in real-time, in a shared moment. In a recent review, results of 512 preterm infants among 15 recent clinical trials using live and recorded maternal voice interventions showed fewer cardiorespiratory episodes and significant improvement of physiologic and behavioral conditions (Filippa et al., 2017).

Examining the mother’s voice, and recordings of her heartbeat sounds have shown auditory cortex improvement (Webb et al., 2015), at 1 month in 40 infants born extremely prematurely (between 25- and 32-week gestation). Newborns were randomized to receive auditory enrichment in the form of audio recordings of maternal sounds (including their mother’s voice and heartbeat) or routine exposure to hospital environmental noise. They were shown to elicit strengthened auditory plasticity at 1 month compared to their controls. This study again used recorded sounds that were static, and not meeting the meter or condition of the premature infant’s signals in the here and now. However, the organized sounds (music) and familiarity rendered better outcomes when compared with noxious hospital noise.

A recent study of five infants with congenital heart disease explored entrainment on the physiologic measurements of withdrawal through live singing and guitar accompaniment. This single case withdrawal pilot study examined the effects of music therapy “entrainment” on heart rate, respiratory rate, blood pressure and oxygen saturation rate, of five infants suffering from congenital heart disease, in the cardiac intensive care unit. Receiving music therapy “entrainment sessions” before and after heart surgery, and consistently, 3–5 times a week for up to 3 weeks, their physiological measures were recorded every 15 s after the music therapy intervention began, until 20 min after the intervention was complete. Although the outcomes showed improvements when “entrainment” was used from baseline to follow up, the songs implemented, when not informed by parents did not necessarily consider range, melodic content, nor musical elements. The entrainment was matched with the guitar accompaniment (many strings) rather than the singing (single-toned phrase). A metronome was instituted, which produces a static rhythm, likely not providing sensitivity to the actual shifts of heart rhythms that one would advantageously follow to match the infants’ rhythmic heart rate changes moment by moment (Yurkovich et al., 2018). Even so, the study had an 80% success rate, whereby four of the five infants experienced decreases in average heart rate and respiratory rate and improvements in the derivative of the heart rate signal as well.

### Music and Executive Functioning

Studies addressing very early exposure to music in (extremely) preterm born infants and a possible outcome-transfer to long-term effects on cognitive functions such as EF or learning are rare. Therefore, it is crucial to understand *music* itself and how it affects, touches and possibly influences brain and cognitive development, in a broader context. Being able to trace musical stimuli through the brain and linking this activity to a possible cognitive effect provides a means toward understanding neural development across the life span. Research on the development of the human brain has indicated that the brain reaches its full level of maturity at an age of around 30 years (Tomlinson, 2015).

Within these crucial years of development, the frontal lobe is one of the last to reach its full maturity (Tomlinson, 2015). With the prefrontal cortex being the primary seat of EFs, correlated with a possible influence of music (a combination of listening, playing and improvising), this area has the potential to influence the developing neural structures through the very complexity of the music itself and the conditions within which it is provided.

Processing music engages long fiber tracts in the brain and overlaps with regions responsible for EFs, and we expect a stimulation of EF when perceiving and performing music (Klein et al., 2016). Understanding the influence of music on EF can, in turn, provide insight into pathologies later in life, which manifest in a form of executive dysfunction. Music therapeutically informed interventions hold the potential, to stimulate the brain, supporting processes of neural plasticity, and can offer an enhanced start into life for (extremely) prematurely born infants, who have entered this world with so often physical and cognitive disadvantages stemming from birth trauma and/or deprivation.

### Music Learning and Brain Function

Early exposure to music can have strong influences on cognitive reserve and development later in childhood (Jaschke, 2019). As there is limited research on the development of EFs in preterm born children (regarding their age for neuropsychological testing) we have reverse—engineered out arguments from the literature which indicate improvements in executive functioning in relation to music training and exposure (Moreno et al., 2011; Degé et al., 2011; Jaschke et al., 2018a,b). This approach affords the tracing back of steps potentially contributing to neural development as informed by neural plasticity, utilizing large published data sets; a method that borrows from historical and anthropological research. We can, therefore, build the argument that by understanding EFs in “neurotypical” or so-called healthy populations we can trace this development back to influences shaped in an NICU environment (Sa de Almeida et al., 2020).

Frontal brain regions mainly process anticipation and expectation and the execution of musical thought during the event of listening and execution. Furthermore, they relate to music improvisation, which also relates to the amygdala and the hippocampus. Of note is the dorsolateral prefrontal cortex (dlPFC), which plays a crucial role in improvisation as well as in learning and memory, connecting deeper brain regions with frontal regions *via* the thalamocortical-thalamic loop. When it comes to music learning and its likely associated executive functioning, it is crucial to examine the difference between music listening and music playing. Both listening and especially playing music, activate a wide array of brain areas. These neural activity networks, however, should be linked in kind to cognitive processes. Understanding the differences between mere exposure to music and playing music, within a collaborative signal-reading environment, can have a significantly different outcome, especially as studies propose causality between music and far transfer domains (Jaschke et al., 2018a).

Linking back to prematurely born infants, to have a deeper understanding of the impact of musical learning and its capacity to affect learning in neurologic development, it may be useful to distinguish four modalities of music participation: (1) passive listening; (2) active listening; (3) music-making; and (4) improvisation. All four, even though consecutively building upon each other, translate uniquely and distinctly to a possible effect on cognition and should, therefore, be considered as influential when analyzing the effects of music on brain function, emotion and behavior.

In premature infancy, the listening conditions might translate well within a quiet or active alert state context, and the musical vocalizing might translate to a contingent singing (Malloch et al., 2012; Shoemark, 2017) or infant-directed singing (Mehr and Krasnow, 2016) exploration. In such contexts, the in-the-moment improvisatory experience might be indicative of a developing repartee and one whereby the music therapist is singing back the premature infant’s vocalization, strategically on the exact pitch of the infant’s tone. Eventually, the premature infant might absorb the vibration percept and create a new tone or an interval of two tones outside of the tone of the formerly set condition. This may unfold without prompt and occur seemingly suddenly, such as a crying sound with accented phrasing, or a comforting sound wherein the rhythmic meter of the plosive mouthing sounds indicates exploration (Loewy, 1995).

## Musical Parameters

### Timing

A recent study of 35, <32 weeks GA neonates revealed that those who listened to 8 min of pre-composed music five times per week, based on nursing assessment of developmental need (assessed on a neonatal behavioral assessment scale), timed to be offered at the moment and distinctly related to need of sleep, wakefulness-interactive, or at a time of alertness, such as prior to feeding, led to functional brain architectures (shown through fMRI) that were more similar to those of full-term newborns (Lordier et al., 2019). This provided their evidence for a “beneficial effect of music on the preterm brain.” This example of identifying the timing of a music intervention is quite indicative of sensitivity to best practice needs and might provide a unique platform for the inclusion of the infant’s state and readiness for music, suggestive of a dynamic aspect of signal reading and its importance when providing intervention for the music applied.

However, the music intervention, and its meter and instruments “composed of a soothing background, bells, harp, and punji (charming snake flute) five times per week from a GA (gestational age) of 33 weeks until the MRI” may not provide aspects related to the most efficacious provisions of the music itself. Unfortunately, readers are not provided with the music applied-and when one seeks to gather the evidence and arrives at the website^1^, the original music provided for this referenced study is not easily found.

Even if it were easily accessed, as a link on-line, because it is an obstructive variable and one that is not reliant on the infants’ vital signs, it is likely not best-indicated. Utilizing a recording, the conditions of timing, so central to a preterm infant’s neural plasticity cannot be sensitively provided as recorded music does not provide a platform for the entrainment features most critical to the infant’s neurologic brain effect. The results for the most ultimately safe and nurturing environment might include the mechanism of entrainment, whereby the parent and/or music therapist (whose training in NICU music therapy) is inclusive of the assessment of the infants’ vital signs and whose timing of intervention is inclusive of the rhythm and melody relevant to the parents’ cultural background/preference, or adapted to be inclusive of musical elements related to these vital conditions. Such cultural aspects necessarily determine whether the music that may be best indicated is meter-less, such as, for example, an Indian raga, or a classically formatted work, such as a baroque piece, or soft rock-set, or folk song, perhaps phrased in 3/4 or 6/8 as a ballad, or in lullaby style. Knowing the music and composers that the infant heard *in utero* and presenting this music live, and eventually constructed within a suitable framework that parents can utilize as their “song of kin” (Loewy, 2015) may ensure continuity of care (by parents, in NICU, and at home post-discharge), with enhanced reliability that might influence the potential for bonding as an incentive for what some researchers have studied to be a “neurologic brain effect” in premature infants. The case for timing of when music is provided, and the timing of the music itself (time signature), and how it is applied (entrained) within its natural meter of culture may be the best-indicated elements one considers for making music therapeutic interventions that seek to strengthen the neurological development of plasticity in premature infants.

### Timbre

The timbre element of sound and music is something rarely discussed in health music applications (such as neurology, music medicine and music therapy). Two innovative NICU studies using recorded music did include the actual womb-sounds of the placenta, combined with female voices. With attention to timbre, these studies incorporated a music program inclusive of the sounds of an intrauterine maternal pulse along with synthesized female voices at 65 dbs. Created by Fred Schwartz, an anesthesiologist, the program was named “Transitions” (Placenta Music, Inc., Atlanta, Georgia). The first study of this music’s effects included four premature infants with Bronchopulmonary dysplasia (BPD) on continuous ventilator support. Each infant was exposed to three different 15-min conditions: (1) music played *via* a Somatron mattress; (2) music played *via* a recorder at the foot of the infant’s crib; and (3) silent isolation room condition. This study included providing music immediately following suctioning. The conditions were counterbalanced and repeated so that each infant received 18 interventions in the following order ABC-BCA-CAB-(etc). They looked at heart rate, oxygen saturation, arousal state, facial expressions, limb movements, and autonomic states all within the 13 s (with 7 s to recover before starting again), every 15 min for 18 trials. The time period of the data collection ranged from 8 to 21 days, every 13 s within a 15 min period for 18 trials. They collected in total 4,860 pieces of data per infant. Oxygen saturation and sleeping time improved significantly during the interventions (Burke et al., 1995).

The second study utilizing Schwartz’s placenta soundscape program was also incorporated in another infants-as-their-own measurement design (Chou et al., 2003). In 30 premature infants with respiratory distress requiring endotracheal intubation and ventilation and endotracheal suctioning, this second trial showed statistically significant higher oxygen saturation during recovery periods compared to the controls, who did not receive the “Transitions” soundscape. Statistical significance was reflected in the period oxygen saturation was achieved, with quicker recovery time when this music was applied. This procedure has potentially disturbing side effects such as dysrhythmias, oxygen desaturation, cerebral blood flow fluctuations, and laryngospasms. Familiar timbres can increase tolerance and build resilience. The potential of watery timbres, reminiscent of womb sounds and their effect on vital sign enhancement should not be overlooked.

Studies of older infants have shown that the mother’s voice, is recognizable to the infant who has heard it distinctly and repetitively in the womb pre-birth (Thompson and Trevathan, 2009). Greater attention taken has achieved the conditions of timbre that replicate a womb-like timbre experience inclusive of water or breathing sounds. These are effectively metered to the premature infant’s rate of breathing. This can occur during kangaroo or skin to skincare, whereby a parent holds their premature infant over their heart (on the left side, Salk, 1973), and can readily pay attention to their infants’ breathing patterns.

The perception of timbre, recruits multiple brain areas, including regions in the frontal lobe, the thalamus—as central relay and multisensory integration region, the hippocampus, amygdala and especially the planum temporal and the temporal plane (Jaschke, 2019). The neural function of perceiving timbre is a combination of pitch decoding, pitch identification, the spatial location of sound and the “color” (warm, cold, squeaky, round) of the perceived sound.

An audible “ah” sound will not only provide a timboric effect of vibration but additionally will offer a means of breath connection, particularly if the parent or music therapist entrains the application of their self- breathing to the meter of the infant’s breathing., as in “tonal-vocal holding” (Loewy et al., 2013). One early study inclusive of entrainment (Ingersoll and Thoman, 1994) included a devised “breathing bear” contraption that could reflect the breathing rate of the infant in proximity. The study included 36 premature infants; half were randomly assigned the breathing bear device vs. the control, a breathing bear device that did not entrain breathing sounds. At 35 weeks, the intervention infants showed slower and more regulated respiration during quiet sleep. At 45 weeks, these same infants showed more quiet sleep and less active sleep. At both ages, only the intervention infants showed a correlation between respiratory regulation and the amount of quiet sleep (Ingersoll and Thoman, 1994). Sleep regulation is a critical mechanism of strong neurologic function that can be influenced by music (Loewy, 2020).

The findings show an effect of positive neurobehavioral development. The author’s credit “entrainment” effects related to optional stimulation. Central to this effect was the reflecting of the infant’s own biological rhythms activated and directed by the infants’ rhythms. Of note, this timbre sound was a water sound set by a machine. It is likely that when a live human being is brought into the response, the essential interplay lays a foundation for not only neurological function but as one of attachment as well.

### Repetition: Predictability as Enhancement in Neonatal Music Therapy

The human brain as it formulates structure in the womb is reliant on the rhythmic conductor of another; that of the mother’s heartbeat. This “conductor” is variable, and the fetus necessarily regulates growth in accordance with a wide range of meters based on the mother’s activity level.

The practice of ritual in patterning music and rhythms of daily life inclusive of the sleep-wake cycles of developing infants is critical (Loewy, 2020). It is known that infants recognize familiar voices and melodies, and language patterns. Repetition, in particular as part of a music gestalt, provides space for interaction and safety within the music’s spaced cue, as in a breath before a downbeat, or within a final repetitive cadence.

It is best practice to engage premature infants with music that has predictability. One study showed that young infants engage more rhythmically to music when compared to speech and that the cued timing related to the effectual mood of songs vs. speech was linked to greater rhythmic coordination (Zentner and Eerola, 2010). A former study (Loewy et al., 2005) comparing chloral hydrate with parents’ songs of kin arranged in lullaby format, incorporating repetition, lead toward a quicker, more profound sedation effect than pharmacologic sedation for infants and toddlers requiring EEG.

### Melody-Simplicity of Range and Recognizable Intervals

It is important when instituting music to seek the melodic organization and sequences of songs that are familiar to the neonate. If parent/s are unavailable, the range of the mother and father’s voice is good to render when providing interventions (eg soprano, alto, tenor, baritone, bass). The “song of kin” (Loewy et al., 2013; Loewy, 2015) is inclusive of a model that assists parents in creating a natural, easily accessed sung melody, and most particularly one that has meaning for the parent. The song’s significance and relevance can be part of a music psychotherapeutic process. It does not have to be a melody that the neonate heard *in utero*. It does not have to be related to spiritual or historical aspects one the infants’ family. The song should aim to be a favored song-culturally relevant to parental preference, and the melody can be entrained and best applied with a simplified single-line matching of sung vocal phrasing. Accompaniment, if and when applied, can be minimal. A Capella is well suited for neonates.

The primary auditory cortex and secondary auditory cortex are the primary preceptors of the incoming auditory information. Wernicke’s area and Heschl’s gyrus, process mimicking and associations, together with pitch intervals and melody (Jaschke, 2019). Music therapy literature has advised on important considerations to keep in mind when implementing best practices of musical melodies for neonates. Notwithstanding cultural nuances, it is useful to consider aspects of caution and simplicity when developing melodies with infants and caregivers: “Slow tempo, simplicity, minimal number of instruments and harmonics, quiet and stable dynamics: decibel levels not greater than 60–65 dB (A-weighted scale), repetition and consistency; rocking meters, one-octave tonal range maximum, unidirectional melodic contours, with limited changed in pitch direction; emphasis on descending tones to engage relaxation response (Stewart, 2009b, p. 127).

### Song of Kin: Affective Culture as Attachment Incentive

Perceiving, processing and executing music recruit numerous neural areas. In combination, the varying facets of music such as rhythm, melody, and timbre are projected to distinct areas of the brain, which decode the stimuli to create what we understand as music. These areas, however, are not exclusive to music. The temporal, as well as frontal lobes that are equally involved in language and arithmetic, are also overlapping within the music-related regions (Jaschke, 2019). Each of the regions includes its own interpretation of the task at hand and therefore stimulating these, increases more than the actual understanding of the task, but can be transferred to other mental exercises such as an increase in empathy or working memory, which share overlapping regions.

The “song of kin” importantly, it is a song that is set to a simple construct, whereby a holding condition or lull (such as 3/4 or 6/8) is implied through an easily accessible form for the parents. It is provided as a developmental tool inclusive of attunement (Bowlby, 1998; Ainsworth, 1989) strategies in the growing relationship between parent and infant. It can be used when an infant is seeking connection-so as to build attachment, in an improvisatory fashion, for instance, such as in the key the infant is cooing within (melodic entrainment). It can be used to calm when an infant is fussy (breath entrainment). It can be used as a ritual, providing the safe assuring conditions of sleep (looping and repeating the cadence to sustain a “cadential effect” (Loewy, 2009, 2020). In the instance of enhancing sleep, the lyrics can be removed, and vowels will elongate melodies and suggest a slowing down of the mind-body-attention.

With so many play songs and activating conditions on the current market related to musical circumstances available for infants and parents, it is perhaps hard to believe that the most nurturing source and use of music can emulate from the parent uniquely, and be formulated to fit the conditions of the infants’ ever-changing physiological state. This was highlighted in a recent study on vocalizations of parents and its influence on eliciting sound-making in preterm infants (Caskey et al., 2011).

As infants do not speak, touch and emotional closeness expressed through vocalizing can forge the seeds of trust and musical repartee (Goulet et al., 1998). The absence of such opportunities within the critical first days of life can increase prenatal anxiety in the NICU (Franck et al., 2005). As musical expression enhances participatory and opportune moments for inter-relating, enhancing a parent’s confidence is essential for bonding. Maternal sensitivity in mothers with preterm infants is less optimal when compared with full-term controls (Forcada-Guex et al., 2006) and early separation from an infant at birth in one study was related to an increase in parents’ NICU-related stress (Franck et al., 2005).

Attachment can be the most intimate and personalized condition of mutual interplay (Ainsworth, 1989) when it is enriched with musical conditions that are personalized. Using the infant’s name and creating circumstances whereby a parent’s singing becomes strengthened and more intense within a response (Loewy, 1995, 2015), such as a silent moment and singing a tone at the same pitch as the infant, or hiding the face and finding it when the infant creates a sound are part of a personalized connecting music soliloquy that prolongs interplay and sustains attention and expectancy factors which are the seeds of brain development.

This is not an instantaneous condition. The experienced clinician or therapist with advanced training deemed fit to work with parents understands that fragile infants have fragile parents (Zimmer, 2019). It is therefore imperative that the music is not simply implemented as a condition that a parent is told is good for the infant. Work in music therapy with mother (Arnon et al., 2014), fathers and families can lend integral inclusion factors that will take a critical community role within developing families (Ettenberger, 2017).

Als et al. (2012) were among the first to follow preterm infants who participated in an individualized developmental approach to neonatal care-currently named the Newborn Individualized Developmental Care and Assessment Program (NIDCAP). The infants provided with NIDCAP showed improved electroencephalogram coherence and more mature frontal brain structural development as evidenced by magnetic resonance imaging at term-corrected age. There were improved neurobehavioral outcomes at 2 weeks and 9 months corrected age (Als et al., 2012).

Moreover, the RBL model includes the parent prong as one of the most essential treatment areas in working with families in the NICU. In playing for and playing music with parents who experience the circumstances of premature birth, opportunities for soothing and nurturance encourage easeful feelings of release and eventually of empowerment (Haslbeck and Costes, 2011). Acknowledgment of the difficult circumstances can circumvent rage, shame, doubt and self-mistrust that are common with premature birth. The provisions of music therapy for parents will likely lead to better outcomes for their capacity to use music in their new relationship with their infant (Loewy, 2015). The effects of trauma on the premature infant in terms of its implications within the developing relationship with the parent/s (Als, 2009) is an understudied aspect of arrested or delayed capacity of neuroplasticity (Stewart, 2009a). Stewart addressed the potential traumatic experiencing for infants and parents and recommended the utilization of a preventative music therapy treatment, inclusive of staff, and based on resiliency and renegotiation principles. The six phases of the model are: (a) stabilization; (b) self-regulation; (c) integration of experience/resolution of traumatic memories; deconditioning; (d) establishment of secure social connections: repair and/or development of effective attachment and reciprocity; (e) accumulation of restorative emotional experience; (f) future planning: development of self-care plans and goals (Stewart, 2009a).

Working with trauma in the NICU requires advanced training, such as the incorporation of Peter Levine’s Somatic Experiencing, or of Stephen Porges’ Polyvagal theory. Aspects of these orientations have been incorporated into the three prong (First Sounds, 2016) music therapy advanced training for NICU music therapists.

## Conclusion

The first 1,000 days from conception to delivery are among the most important periods in the development of an individual. During this time, the infant brain is rapidly developing and is extremely sensitive to environmental influences. For preterm infants, born before a gestational age of 37 weeks, and specifically, those born very preterm (i.e., before 30 weeks’ gestation), the period that should have been spent in the womb, is interrupted, often suddenly. Preterm infants often undergo trauma, that may be experienced by their parents (Aagard and Hall, 2009) as they, at the same time, are subsequently admitted to the NICU, where they face many challenges. Among these challenges is the stress of physical and sensorial influences coupled with maternal separation.

Music as an often complex and cognitively demanding modality can be over-stimulating particularly when considering the fragility of the neonatal brain developmentally. Therefore, a conservative “less is better” minimalistic approach to the music instituted is recommended. Opportunities for music therapy assessment that implement one stimulus at a time, with sensitivity to rhythm, decibel level, proximity to the infant (at mid-line to encourage fetal positioning) is indicated. Entrainment conditions where the parent/therapist is trained to follow the infant’s cues and clues, with breath (air/water timbre qualities), and melody, with small, repetitive short phrases comprised of the cultural sequences and sung by familiar voices have been substantiated (Loewy, 2015; Mondanaro et al., 2016).

Opportunities to treat fragile parents directly with music therapy relaxation techniques may provide a wealth of physiological sensorial conditions that in turn, may be sensitively and naturally translated to their infants. Adherence to reading the signals of the neonates’ readiness or disengagement, inactive alert, quiet-alert, or sleep state preferences will provide key information related to the most opportune times for live music that can stimulate interactive activity, or alternatively induce restful transitions for sleep that will ultimately enhance neonatal brain function. The clinical applications of music are best integrated when elements of decision-making related to musical choices are substantiated by cultural, psychological and neurological mechanisms. In this way, clinicians, caregivers and researchers alike can incorporate elements of music that will assist in regulating, enhancing, adapting and fostering the music to meet the cues and clues of the ultimate conductors—the premature infant and parent/s we serve.

## Author Contributions

JL conceived the manuscript concept and created the outline, authoring the introductions and body-support of the hypotheses, based on research on neonatal music therapy in Mount Sinai Beth Israel. AJ co-contributed to the neuroscience theory, enhancing aspects related to music mechanisms in neural plasticity.

## Conflict of Interest

The authors declare that the research was conducted in the absence of any commercial or financial relationships that could be construed as a potential conflict of interest.

## Footnotes

^1^http://vollenweider.com/en
